# Shear wave elastography in assessing stiffness and volume of varicocele-affected and normal testes: a systematic review and meta-analysis

**DOI:** 10.1186/s12610-025-00282-1

**Published:** 2025-08-26

**Authors:** Lihong Wang, Xiaoqi Huang, Yinman Ding

**Affiliations:** Department of Urology, Xuancheng People’s Hospital, Xuancheng, 242000 China

**Keywords:** Varicocele, Shear wave elastography, Testis, Stiffness, Volume, Varicocèle, Elastographie par Ondes de Cisaillement, Testicule, Rigidité, Volume

## Abstract

**Background:**

Varicocele (VC) is a significant contributor to both primary and secondary male infertility. VC can lead to progressive, time-dependent damage to testicular function, as demonstrated in both animal and human studies. To assess testicular tissue alterations, we conducted the first systematic review and meta-analysis on the effects of VC on testicular stiffness and volume. A literature search was conducted in June 2024 without language or geographic restrictions. The search included the databases PubMed, Embase, and Medline. We reviewed the literature to identify all published clinical trials using shear wave elastography (SWE) to evaluate the stiffness and volume of varicocele-affected and normal testes. The reference lists of the retrieved studies were examined. We conducted a systematic review and meta-analysis.

**Results:**

Seven articles, encompassing 1118 testes, were selected from a pool of eighty-seven. The analysis revealed that the stiffness of varicocele-affected ipsilateral testes was higher than that of normal testes (*p* < 0.05), and the stiffness of normal testes was higher than that of contralateral testes (*p* < 0.05). Regarding volume, the varicocele-affected ipsilateral testes were smaller than the contralateral testes (*p* < 0.05), with no significant difference between the contralateral and normal testes (*p* > 0.05).

**Conclusions:**

This meta-analysis indicates that VC impacts the stiffness and volume of both testes. SWE is an effective technique for assessing testes affected by VC, but a large-scale, multicenter randomized controlled study is needed for further confirmation.

## Résumé

**Contexte:**

La varicocèle est un contributeur important à l’infertilité masculine primaire et secondaire. La varicocèle peut entraîner des dommages, progressifs et dépendants du temps, de la fonction testiculaire, comme l’ont démontré des études animales et humaines. Pour évaluer les altérations du tissu testiculaire, nous avons effectué une revue systématique et méta-analyse sur les effets de la varicocèle sur la rigidité et le volume des testicules. Une recherche bibliographique a été effectuée en juin 2024 sans restriction linguistique ou géographique. Ont été incluses les bases de données PubMed, Embase et Medline. Nous avons examiné la littérature pour identifier tous les essais cliniques publiés utilisant l’élastographie par ondes de cisaillement pour évaluer la rigidité et le volume de testicules atteints de varicocèle et de testicules normaux. Les références bibliographiques des études récupérées ont été examinées.

**Résultats:**

Sept articles, comprenant 1118 testicules, ont été sélectionnés parmi un pool de quatre-vingt-sept. L’analyse a révélé que la rigidité des testicules ipsilatéraux affectés par les varicocèles était supérieure à celle de testicules normaux (*p* < 0,05), et que la rigidité de testicules normaux était supérieure à celle des testicules controlatéraux (*p* < 0,05). En ce qui concerne le volume, les testicules ipsilatéraux affectés par la varicocèle étaient plus petits que les testicules controlatéraux (*p* < 0,05), sans différence significative entre les testicules controlatéraux et des testicules normaux (*p* > 0,05).

**Conclusions:**

Cette méta-analyse indique que la varicocèle a un impact sur la rigidité et le volume des deux testicules. L’élastographie par ondes de cisaillement est une technique efficace pour évaluer les testicules affectés par la varicocèle, mais une étude contrôlée randomisée multicentrique à grande échelle est nécessaire pour une confirmation supplémentaire.

## Background

Varicocele (VC) is a vascular disorder characterized by the abnormal twisting and dilation of the pampiniform plexus veins within the spermatic cord. It is commonly observed in young adults, predominantly on the left side, and can cause male infertility, testicular hypofunction, pain, and discomfort, making it a leading cause of male infertility. Approximately 15% of adult males in the general population are affected by VC. While some patients can conceive without intervention, primary infertile males have a 35%−44% incidence of VC, which increases to 45%−81% in secondary infertile males [1]. This suggests that VC is likely a progressive condition requiring appropriate treatment [2]. 

VC can be identified through clinical examination. Color duplex ultrasound (RDUS) is another method used to confirm the diagnosis. Although RDUS is effective for diagnosing VC, it cannot detect tissue degradation before testicular atrophy.[3] Recent advancements in ultrasound (US) technology have introduced new perspectives on the anatomical and functional assessment of testicular tissue [4]. Shear wave elastography (SWE) is a simple, rapid, and feasible imaging modality. It measures tissue stiffness or"elasticity"by analyzing the speed and propagation pattern of shear waves through the tissue. Soft tissue produces low-speed shear waves, while higher speeds are observed in hard or stiff tissue. It is used in assessing the breast, prostate, thyroid, and liver, particularly for evaluating focal lesions and neoplasms [5–8]. Testicular tissue elasticity has been studied using SWE;[9] the results remain controversial. Therefore, we conducted the first meta-analysis to investigate the contentious effects of VC on testicular stiffness and volume. Our meta-analysis aimed to assess changes in stiffness and volume between varicocele-affected and normal testes.

## Methods

### Search Strategy

We conducted a literature search in July 2024 using the PubMed, EMBASE and MEDLINE databases. We scrutinized the reference lists of the included studies to further choose the more relevant articles and abstracts. We used the following search terms: varicocele, testis, stiffness, volume and elastography.

### Inclusion Criteria

Clinical trials that met the following criteria were included: (1) a study design in patients with VC group and control group; (2) the study provided accurate data that could be analyzed and included two objects of study (testicular stiffness and volume) between VC group and control groups, respectively; (3) the outcome reported as the mean and standard deviation or the median and quartile; and (4) the full text of the study could be accessed. If these inclusion criteria were not met, then the study was excluded from the analysis.

### Trial Selection

**Fig. 1:**
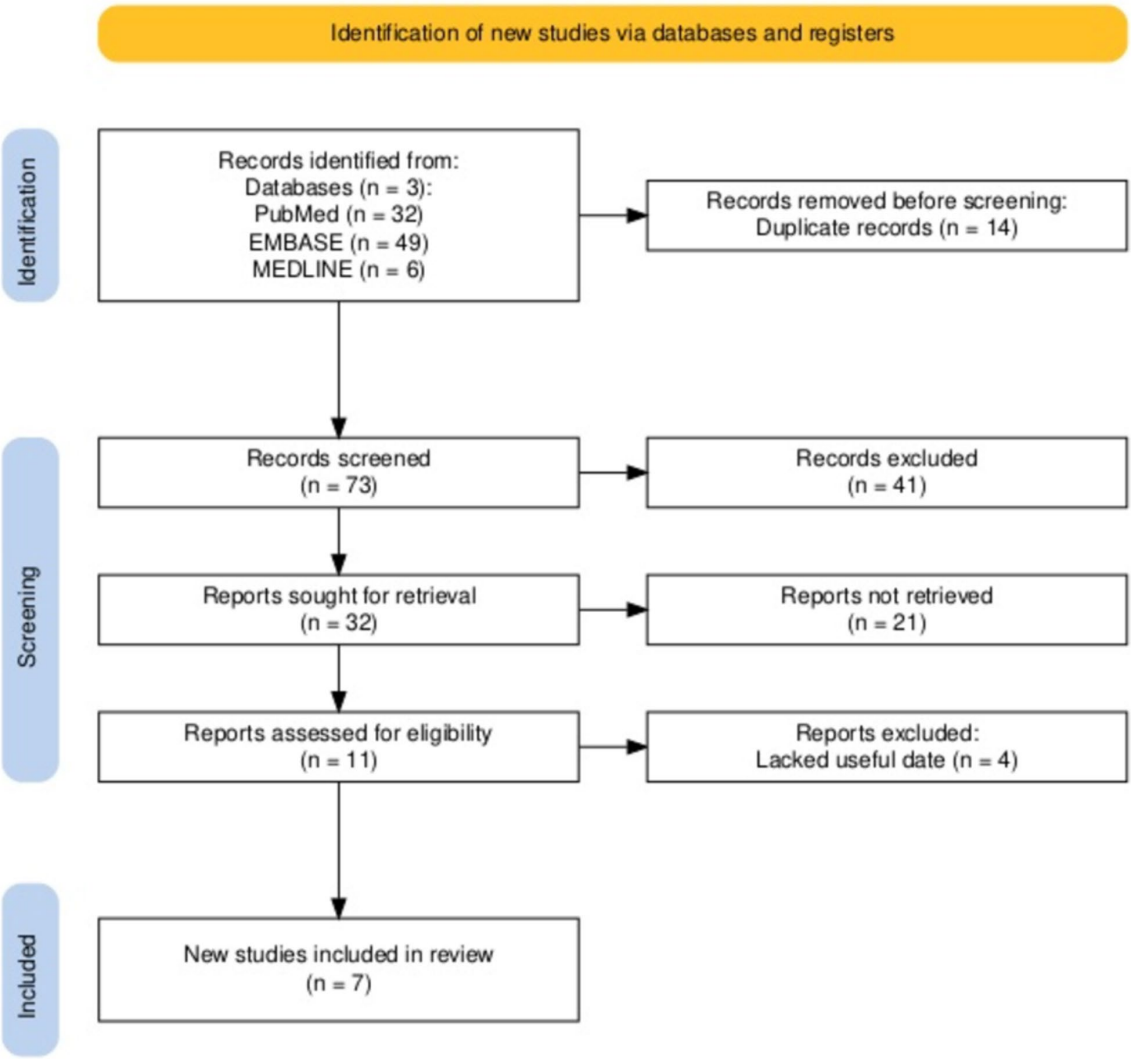
The Flow Diagram of the Study Selection

If the same group of subjects was studied in multiple experiments, each study was included. We made it clear that we did not apply the same data to the studies'findings more than once, even though we tried to include as more as feasible. The most often cited article was included when the same study was published in articles. Every study that was included or eliminated was discussed. A flow diagram of the study selection process is presented in Fig. 1.

### Quality Assessment

The included studies'quality was evaluated by two independent reviewers using the Preferred Reporting Items for Systematic Reviews and Meta-analyses guidelines. Risk factors in research generally cannot be randomized which relates to innate human characteristics or practices, and it is unethical to expose subjects to harmful risk factors. This is true not only of the randomized controlled trials that we included in our meta-analysis, but of numerous observational studies, such as comparative studies and controlled clinical trials [10]. Numerous risk factors that are not amenable to randomization exist in study. Each study's quality was generally categorized into one of the following two groups based on the quality assessment criteria: A study was judged to have a low risk of bias if all quality requirements were met (sufficient); B studies were judged to have a moderate risk of bias if one or more quality criteria were only partially met (unclear). The reviewers'discussions helped to resolve disagreements.

### Data Extraction

The data were extracted and cross-checked by two independent reviewers using a predesigned form, which included the first author’s name, publication year, number of VC and control testes, age, country. Disagreements were discussed by the third person. The primary outcome was the changes in stiffness between varicocele-affected and normal testes. The secondary outcome was volume between varicocele-bearing and normal testes.

### Statistical Analysis

**Fig. 2:**
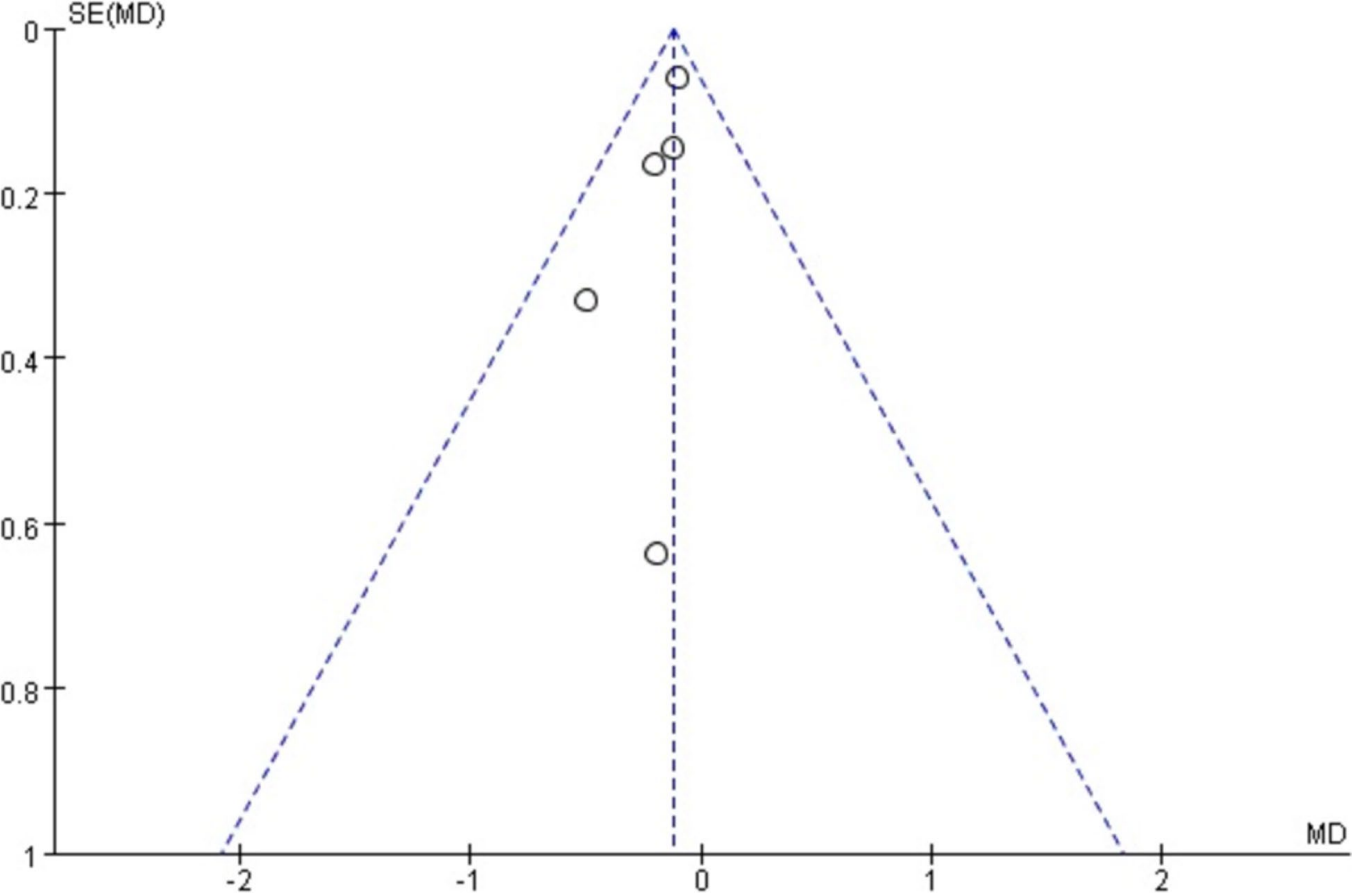
Funnel Plot of the Studies Represented in this Meta-Analysis

Statistical analysis was performed by Review Manager 5.4. Outcomes were expressed as continuous outcomes, including the mean difference (MD) with 95% confidence interval (CI) and.

the p value and odds ratios (OR) for dichotomous outcomes. We used the I^2^ heterogeneity test to quantify the effect of result heterogeneity. If I^2^ was no more than 50%, we chose a fixed effects model. If I^2^ was more than 50%, we chose a random effects model. We used a funnel plot to evaluate the presence of publication bias. A funnel plot of the studies represented in this meta-analysis is presented in Fig. 2.

## Results

### Characteristics of Individual Studies

The database search identified 87 articles that were potential candidates for inclusion in our meta-analysis. Based on the inclusion and exclusion criteria, forty-one articles were excluded after a preliminary review of their titles and abstracts. Twenty-one articles were excluded which did not distinguish between the testes with VC and the contralateral testes. Four articles were excluded due to insufficient data. All seven articles (Baleato-Gonzalez et al., 2023; Erdogan et al., 2020; Fuschi et al., 2021; Jedrzejewski et al., 2019; Mulati et al., 2021; Turna et al., 2020; Yüzkan et al., 2023), involving a total of 1,118 testes, were included in the analysis. The clinical trials in these articles were conducted in Spain, Turkey, Italy, Poland, and China. The number of testes included in each of the seven studies was relatively small, ranging from 13 to 116 testes. The baseline characteristics of the studies included in our meta-analysis are summarized in Table 1.

**Table 1:** The Main Characteristic and Quality Assessment of Eligible Studies

Author,year	Country	Testes with VC group				Contralateral testes group				Control testes group				Quality assessment^a^
		Age (years)	Number	Stiffness (kPa)	Volume (ml)	Age (years)	Number	Stiffness (kPa)	Volume (ml)	Age (years)	Number	Stiffness (kPa)	Volume (ml)	
Baleato-Gonzalez (2023)	Spain	40.4 ± 8.7	19	4.0 ± 0.4	15.8 ± 3.8	38.3 ± 9.3	13	4.0 ± 0.5	16.0 ± 4.3	43.7 ± 11.9	62	4.2 ± 0.7	16.4 ± 5.9	A
Erdogan (2020)	Turkey	28.56 ± 8.95	50	12.61 ± 6.23	13.43 ± 4.64	27.52 ± 9.28	46	9.23 ± 3.23	14.29 ± 3.82	28.79 ± 11.62	104	9.42 ± 4.30	15.27 ± 4.13	A
Fuschi (2021)	Italy	27.44 ± 6.09	82	2.56 ± 0.814	12.83 ± 2.27	27.44 ± 6.09	82	2.075 ± 0.39	17.47 ± 2.35	NA	NA	NA	NA	A
Jedrzejewski (2019)	Poland	14.9 ± 2.2	30	2.61 ± 0.66	13.3 ± 5.6	14.9 ± 2.2	30	2.40 ± 0.61	15.9 ± 6.3	NA	NA	NA	NA	A
Mulati (2021)	China	30(24–33)	67	5.7(4.97–6.51)	8.514 ± 2.985	30(24–33)	67	4.08(3.8–4.25)	13.487(11.422–15.790)	29(28–31)	30	Left:4.29(3.8075–4.385) Right:4.12(3.9925–4.25)	Left:12.331 ± 1.565 Right:14.081(13.180–14.955)	B
Turna (2020)	Turkey	32.81 ± 9.07	Normospermic 29 Oligospermic 29	Normospermic:4.77 ± 1.16 Oligospermic:6.15 ± 1.96	Normospermic:12.84 ± 2.68 Oligospermic:12.44 ± 2.82	32.81 ± 9.07	Normospermi 29 Oligospermic 29	Normospermic:3.52 ± 0.41 Oligospermic:3.81 ± 0.71	Normospermic:13.44 ± 4.07 Oligospermic:15.01 ± 5.44	34.23 ± 9.09	58	3.79 ± 0.94	15.75 ± 4.51	B
Yüzkan (2023)	Turkey	33.4 ± 10.3	66	7.90 ± 2.26	15.2 ± 4.16	32.4 ± 9.7	50	7.44 ± 1.90	15.4 ± 4.58	33.7 ± 12	116	7.94 ± 2.07	16.8 ± 4.30	A

*NA* not available

^a^*A* If all quality criteria were adequately met, the study was deemed to have a low risk of bias, *B* if one or more of the quality criteria was only partially met or was unclear, the study was deemed to have a moderate risk of bias

### Changes in testicular stiffness between varicocele-bearing and normal testes

**Fig. 3:**
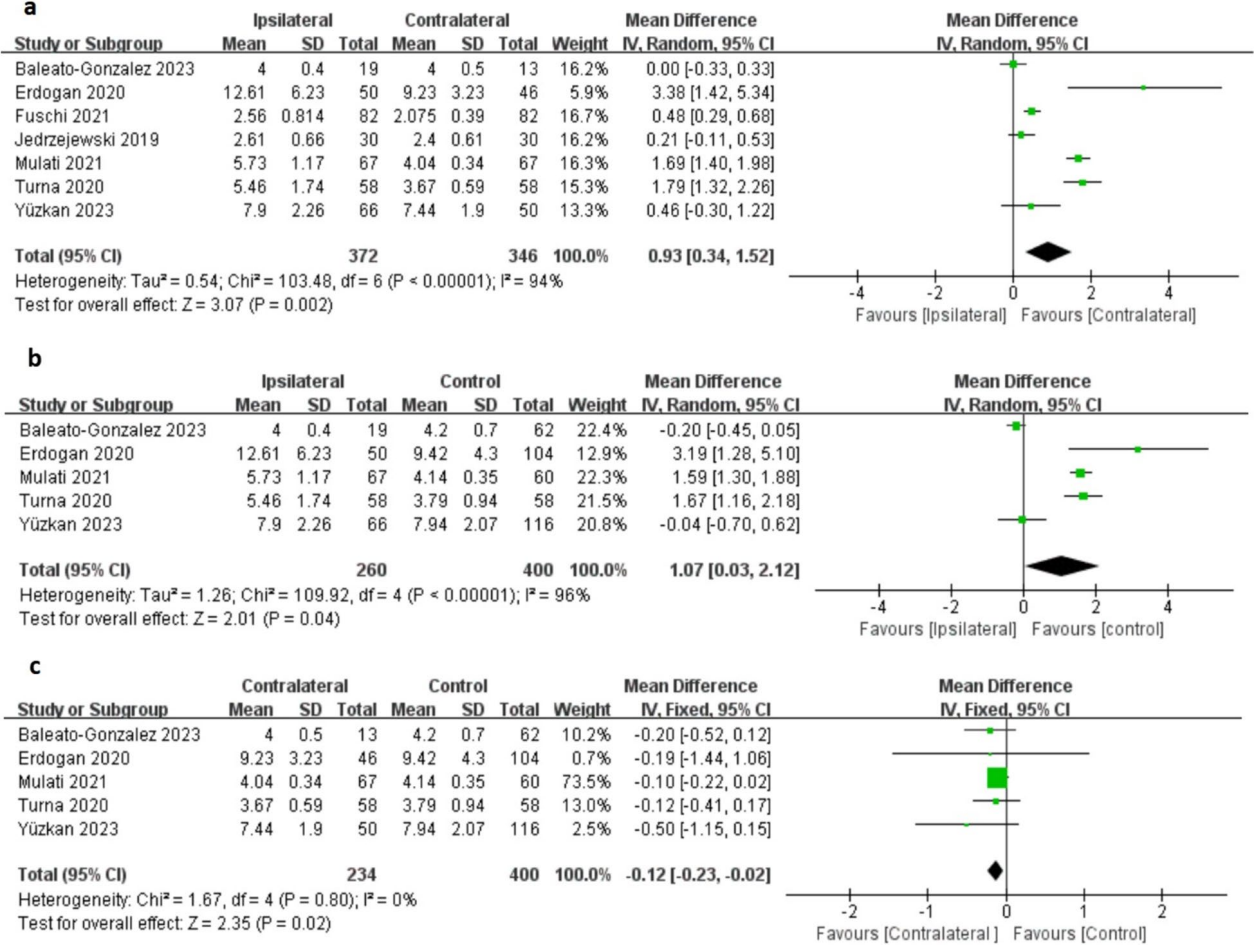
Forest Plots Showing the Changes of testicular stiffness. (**a**) Ipsilateral vs. Contralateral. (**b**) Ipsilateral vs. Control and (**c**) Contralateral vs. Control

Seven studies included data on changes in testicular stiffness, representing 718 testes (372 varicocele-affected and 346 contralateral). The heterogeneity test indicated variability among the trials, leading to the selection of a random-effects model. The pooled mean difference (MD) was 0.93 kPa, with a 95% confidence interval (CI) of 0.34 to 1.52, *p* < 0.05. This result indicates that testicular stiffness increased by 0.93 kPa on the affected side compared to the contralateral side (Fig. 3a).

Five studies provided data on changes in testicular stiffness, representing 660 testes (260 with varicocele-affected and 400 control). The heterogeneity test indicated variability among the trials, leading to the selection of a random-effects model. The pooled MD was 1.07 kPa, with a 95% CI of 0.03 to 2.12, *p* < 0.05. This result indicates that testicular stiffness increased by 1.07 kPa on the affected side compared to the control group (Fig. 3b).

Five studies provided data on changes in testicular stiffness, representing 634 testes (234 contralateral and 400 control). The heterogeneity test indicated no variability among the trials, leading to the selection of a fixed-effects model. The pooled MD was −0.12 kPa, with a 95% CI of −0.23 to −0.02, *p* < 0.05. This result indicates that testicular stiffness decreased by 0.12 kPa on the contralateral side compared to the control group (Fig. 3c).

### Changes in testicular volume between varicocele-bearing and normal testes

**Fig. 4:**
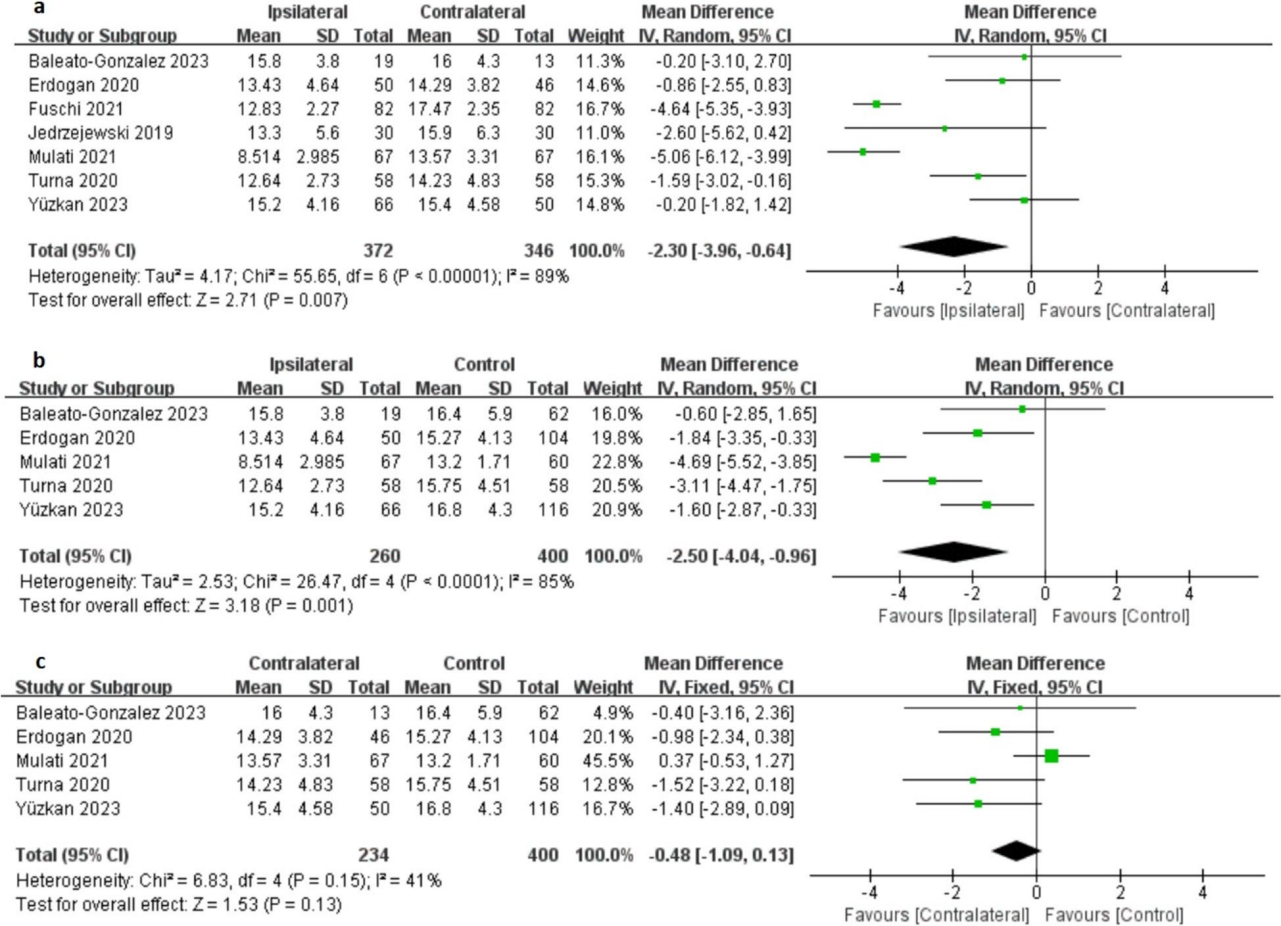
Forest Plots Showing the Changes of testicular volume. (**a**) Ipsilateral vs. Contralateral. (**b**) Ipsilateral vs. Control and (**c**) Contralateral vs. Control

Seven studies provided data on changes in testicular volume, representing 718 testes (372 with varicocele-affected and 346 contralateral). The heterogeneity test indicated variability among the trials, leading to the selection of a random-effects model. The pooled MD was −2.30 ml, with a 95% CI of −3.96 to −0.64, *p* < 0.05. This result indicates that testicular volume decreased by 2.30 ml on the affected side compared to the contralateral side (Fig. 4a).

Five studies provided data on changes in testicular volume, representing 660 testes (260 with varicocele-affected and 400 control). The heterogeneity test indicated variability among the trials, leading to the selection of a random-effects model. The pooled MD was −2.50 ml, with a 95% CI of −4.04 to −0.96, *p* < 0.05. This result indicates that testicular volume decreased by 2.50 ml on the affected side compared to the control group (Fig. 4b).

Five studies provided data on changes in testicular volume, representing 634 testes (234 contralateral and 400 control). The heterogeneity test indicated no variability among the trials, leading to the selection of a fixed-effects model. The pooled MD was −0.48 ml, with a 95% CI of −1.09 to 0.13, *p* > 0.05. This result indicates no significant difference in testicular volume between the contralateral side and the control group (Fig. 4c).

## Discussion

In patients with VC, the pampiniform plexus veins show dilatation, stasis, and increased pressure. Approximately 20–40% of infertile males are affected by VC [11]. VC is a common cause of male infertility, but the underlying mechanisms remain unclear. Potential mechanisms include increased venous tension, elevated testicular temperature, hypoxia, reduced testicular blood flow, androgen deprivation, reflux of toxic and adrenal metabolites into the testes, increased apoptosis, and DNA damage [12].

The effects of VC on testicular tissue, spermatogenesis, and the role of apoptosis remain unclear. A"stress pattern"has been identified in the testicular tissue alterations observed in VC patients. This stress pattern consists of small, immature cells. These cytomorphologic changes resemble apoptotic cells, but are not considered normal [13]. Subsequent research has linked the onset of testicular apoptosis to degenerative changes in germ cells caused by elevated temperatures [14]. Testicular examination may reveal parenchymal atrophy and softening of the testicular parenchyma, possibly due to apoptosis.

Patients with VC often perceive the affected testicle as softer during physical examination. However, actual testicular texture alterations in VC do not align with this perception. Research has shown that VC causes testicular dysfunction and alters testicular hemodynamics. The combined effects of metabolite accumulation from the kidneys and adrenal glands, along with reduced blood flow due to VC, further impair testicular function. Abnormal hemodynamics, toxin reflux deposition, hypoxia, inflammation, and oxidative stress can lead to pathological changes such as testicular microvascular intimal hyperplasia and endothelial cell degeneration. These conditions cause spermatogenic cell shedding, fibrosis, degeneration of surrounding tissues, and interstitial cell edema, significantly impairing the appearance and function of the testes.[15, 16].

Two main types of elastography are in use: shear wave elastography (SWE), which measures the speed of shear waves to create an image of tissue stiffness, and strain elastography, which calculates tissue strain by measuring displacement in response to light pressure [17]. SWE estimates shear wave speed, which is higher in rigid tissues and lower in soft tissues [18]. SWE is a non-invasive technique for assessing tissue stiffness and could be valuable for diagnosing, staging, and treating disorders involving changes in tissue elasticity. Shear wave speed is measured in m/s and kPa [5]. These quantitative elasticity values provide robust diagnostic capabilities. However, one study found that the area under the receiver operating characteristic (ROC) curve for lesions, measured in kPa, was significantly higher than that measured in m/s [19]. SWE is widely used in diagnosing testicular disorders and abnormalities. In recent years, SWE has been used to assess testicular tumors, torsion, infarcts, and VC [20].

In our analysis, we found that the stiffness of the affected testis was significantly higher than that of both the contralateral and normal testes (*p* < 0.05), and the stiffness of the contralateral testis was lower than that of the normal testes (*p* < 0.05). These results suggest that VC increases the stiffness of the affected testis while reducing the stiffness of the contralateral testis. We also evaluated changes in testicular volume, finding that the affected testis had a significantly lower volume than both the contralateral and normal testes (*p* < 0.05). There was no significant difference in volume between the contralateral and normal testes (*P* > 0.05). This suggests that VC decreases the volume of the affected testis without affecting the contralateral testis.

Our results confirm that testicular volume decreases in the affected testis compared to normal testes. However, this decrease is not correlated with the degree of fibrosis, as shown by the lack of correlation between SWE values and testicular volume in our study. Therefore, testicular volume cannot be considered a reliable indicator of parenchymal injury severity. The small sample size may have influenced this outcome. The literature shows inconsistent results regarding whether testicular volume is a reliable criterion for predicting the degree of interstitial fibrosis.[21–24] High SWE values may indicate interstitial fibrosis, potentially reflecting the degree of damage more accurately than testicular volume. Pathological investigations have shown that testes affected by VC exhibit reduced seminiferous tubule diameter, peritubular fibrosis, and Leydig cell atrophy.[25] Although testicular biopsy is an invasive procedure that can evaluate the histological characteristics of parenchymal injury, it is not the preferred approach. Semen analysis can inadvertently indicate parenchymal damage.[26] Our study found significant differences in SWE values between the ipsilateral, contralateral, and normal testes. Affected testes had significantly higher SWE levels compared to both contralateral and normal testes.

Our research suggests that SWE values generally increase with the degree of fibrosis. SWE is a reliable method for assessing variations in testicular tissue stiffness in VC patients. Previous histological analysis in rabbits further supports the potential use of SWE in assessing testicular tissue stiffness.[27] Furthermore, SWE is a valuable tool for evaluating and monitoring the extent, progression, or regression of damage after surgery. Although our study was not confirmed by histopathological examination, SWE was found to be a valuable method for evaluating testicular damage in VC. Larger studies are needed to confirm the effectiveness of SWE in routine VC evaluation.

### Limitations

This meta-analysis included 7 studies involving 1118 testes. The sample sizes were relatively small. Additionally, unpublished studies were not considered in this analysis. These factors may have introduced bias. The primary source of bias was that some included studies were observational, such as comparative studies and controlled clinical trials. Additionally, the outcomes of these studies may have been assessed using different methods. Furthermore, the researchers conducting the trials were different. Finally, potential selection biases may have affected group homogeneity, and the relatively small sample sizes limited the statistical power to identify true associations. Despite the heterogeneity among individual studies, this meta-analysis is crucial for assessing the impact of VC on testicular stiffness and volume changes. Further high-quality trials with larger sample sizes are needed to better understand the impact of VC on testicular function.

## Conclusion

In summary, our study suggests that SWE is an effective technique for assessing testes affected by VC, predicting interstitial fibrosis, and monitoring the severity of parenchymal damage. VC can alter the stiffness and volume of bilateral testes, potentially contributing to male infertility. A larger sample size is needed to confirm the effectiveness of SWE in routine VC evaluation.

## Data Availability

No datasets were generated or analysed during the current study.
